# Diffuse Dermal Angiomatosis of the Breast

**DOI:** 10.7759/cureus.64681

**Published:** 2024-07-16

**Authors:** Pooja Shet, Victoria J Shi, Anand Rajpara

**Affiliations:** 1 Dermatology, University of Missouri-Kansas City School of Medicine, Kansas City, USA

**Keywords:** hypoxia-induced condition, cutaneous reactive angiomatosis, benign vascular proliferation, reactive angioendotheliomatosis, diffuse dermal angiomatosis

## Abstract

Diffuse dermal angiomatosis (DDA) is a rare, benign cutaneous disorder that can affect the breasts. Typically, it presents in middle-aged women and is increasingly associated with various risk factors that involve tissue hypoxia. We report this case of classical bilateral DDA of the breasts in a 56-year-old female patient. This case highlights the association of DDA with hypoxia-inducing risk factors, such as smoking. Management of the hypoxic risk factors resulted in the resolution of the bilateral ulceration caused by DDA in this patient. This case report aims to discuss the etiology, risk factors, clinical manifestations, and treatment modalities commonly used to manage this condition.

## Introduction

Diffuse dermal angiomatosis (DDA) is a benign vascular disorder, previously described as a rare variant of reactive cutaneous angioendotheliomatosis (RAE). More recently, it has been described as a distinct disorder on the spectrum of reactive cutaneous angioendotheliomatoses rather than a variant. Diffuse dermal angiomatosis of the breast (DDAB) is a rare benign condition that is most common among middle-aged, overweight women with macromastia and a history of vaso-occlusive disorders and/or smoking [1,2].

Clinically, patients present with painful, non-healing, reticulated, violaceous, centrally ulcerating lesions or plaques on the breasts. Vascular prominence can also be visualized on the breasts [3]. Lesions can also enlarge over time [4]. We present the case of a patient with bilateral ulceration of the lower breasts triggered by lifestyle factors and family history, with marked improvement after the removal of the inflammatory lifestyle factors.

## Case presentation

A 56-year-old female presented to the dermatology clinic for recurrent, extremely painful ulcerations of the bilateral lower breasts that appeared six months prior. The same ulceration occurred two years prior with a resolution of her symptoms following antibiotic treatment. At a previous facility, a biopsy was performed of her breasts which had shown no histologic evidence of malignancy. She had taken two rounds of cephalexin and clindamycin and used topical mupirocin for this current occurrence with minimal improvement in her symptoms.

The patient’s medical history was notable for hypertension, hyperlipidemia, coronary artery disease, fibromyalgia, obesity, and myocardial infarction four years ago. She had also undergone breast reduction surgery 18 years prior. Her daily medications included baclofen, carvedilol, ezetimibe, gabapentin, hydrochlorothiazide, isosorbide mononitrate, losartan, and rosuvastatin.

The patient, at the time of presentation, was a current tobacco smoker. She had no history of diabetes and her hemoglobin A1C was 5.3% at her last general visit. She denied any other recent illnesses or new medications.

**Figure 1:**
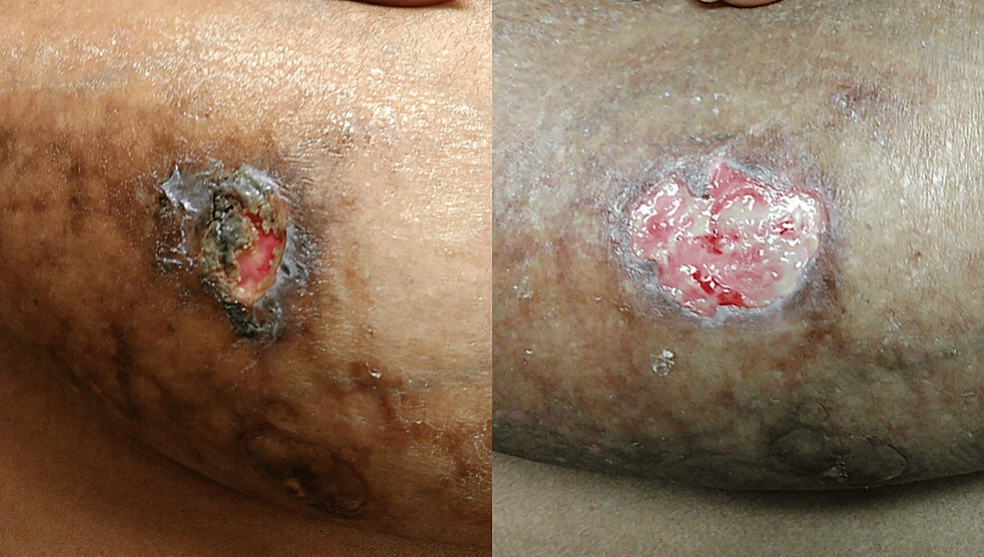
Clinical presentation of the breasts showing ulceration and bilateral reticulated erythematous violaceous plaques.

**Figure 2:**
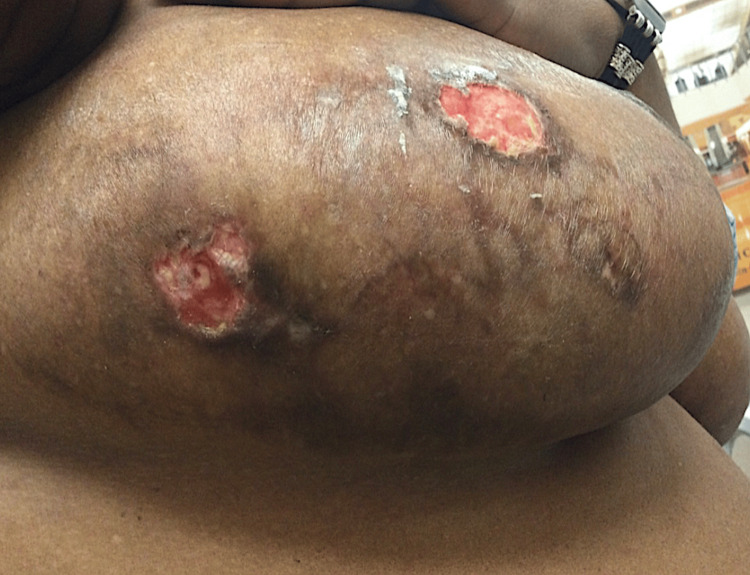
Clinical presentation of diffuse dermal angiomatosis showing superficial ulceration of the right breast.

Physical examination revealed large pendulous breasts with bilateral reticulated violaceous plaques (Figure 1) and superficial ulcerations (Figure 2). No ulcerations on the lower extremities were identified. She reported no fevers, weight loss, or additional constitutional symptoms. A mammogram during this visit was negative for malignancy. A prior tissue culture was negative for growth. Human herpesvirus 8 testing was negative.

The patient was seen by wound care, who instructed her to dress the wound with collagen, silver dressings, and foam. Debridement of the wound was also done at this visit. She returned to the wound care clinic after two weeks and noted extreme pain following debridement. She was then dressed with nystatin/triamcinolone cream to the peri-wound. Silver hydrogel was applied to the wound beds, covered with Xeroform dressing, and secured with Mepilex tape. She was then referred to dermatology.

**Figure 3:**
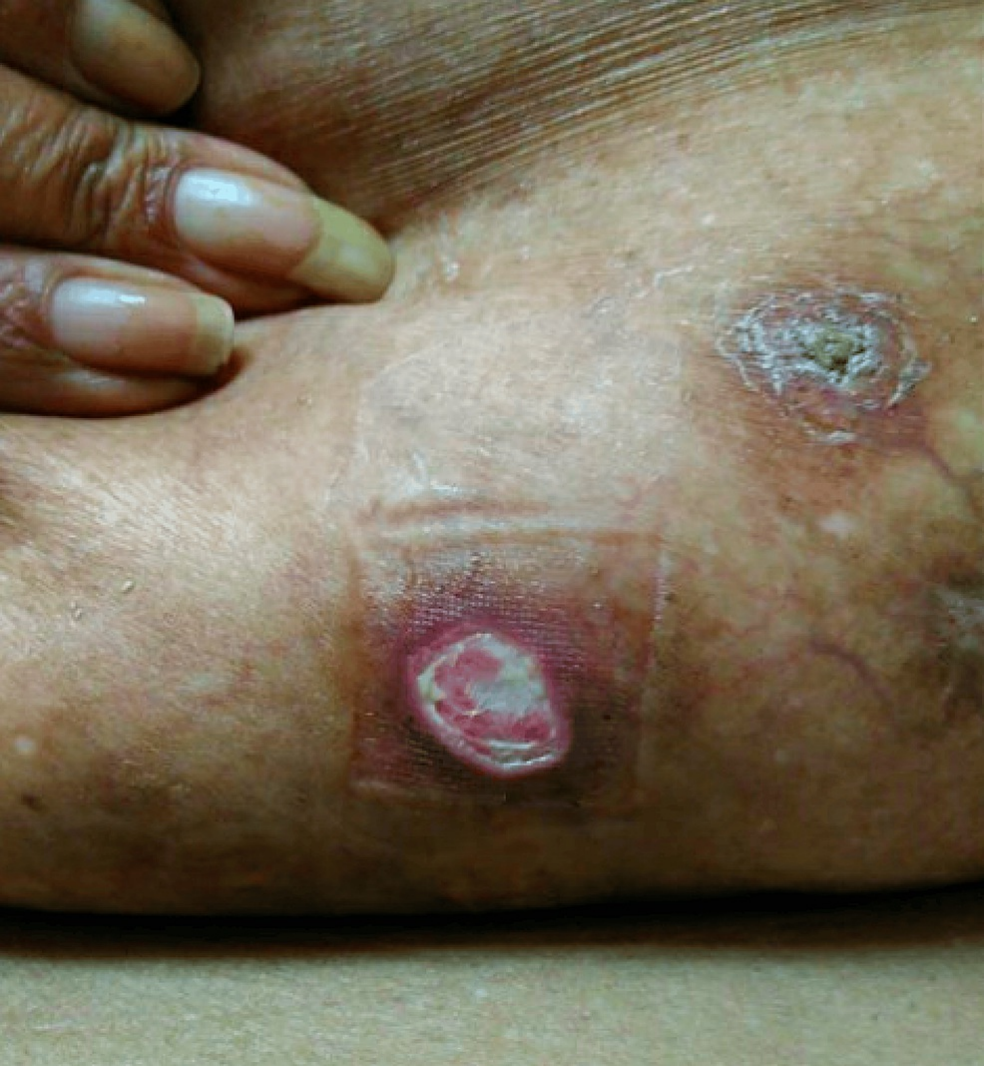
Moderate resolution of ulcers.

She was diagnosed with DDAB based on characteristic visual inspection and patient history. After the diagnosis of DDAB was determined, the patient was educated regarding smoking cessation, weight loss, and control of her hyperlipidemia. She consequently lost weight and stopped smoking. After modification of her lifestyle and adherence to Pentoxifylline 400 mg BID, her ulcers were noted to have resolved and an overall improvement in DDA lesions was seen (Figure 3).

## Discussion

The patient’s history of cardiovascular risk factors and clinical features of ulceration confined to the lower breast region are consistent with a diagnosis of DDAB. Clinically, DDAB manifests with tender, erythematous, violaceous plaques and purpuric papules. Lesions may progress to central ulceration with surrounding tissue necrosis [4]. The lesions seen in DDA are clinically indistinguishable from RAE [5]. The pathogenesis of DDAB is not completely understood. It is hypothesized that prolonged ischemia causes upregulation of vascular endothelial growth factor (VEGF), leading to angiogenesis. Tissue compression and increased venous hydrostatic pressure caused by macromastia can further upregulate VEGF and angioproliferative cytokines.

Recent studies have also suggested that gestational gigantomastia, a rare disorder of pregnancy, can also potentially cause DDAB [6]. Obstructive sleep apnea, commonly seen in obese women, can also exacerbate ischemia and worsen the tissue hypoxia seen in these patients [2]. This patient, specifically, had various vascular disease comorbidities, including hypertension, hyperlipidemia, coronary artery disease, and a history of myocardial infarction, which increased her risk for DDA. DDA has also been seen to be associated with various predisposing comorbid conditions, such as iatrogenic arteriovenous fistula, monoclonal gammopathy, calciphylaxis, antiphospholipid syndrome, advanced atherosclerotic vascular diseases, and vaso-occlusive disorders, such as peripheral artery disease and Takayasu arteritis [2].

Management of DDAB consists of improving the causative tissue hypoxia and ischemia. Patient education regarding smoking cessation, weight loss, and atherosclerotic risk factor control is important to prevent the worsening of the disease. In women with cardiovascular risk factors, further evaluation of the subclavian artery is needed to exclude arterial occlusion. Conservative treatment includes isotretinoin, nifedipine, aspirin, pentoxifylline, or corticosteroids. Bilateral reduction mammoplasty and revascularization are associated with definitive improvement of DDAB in women with preexisting macromastia [2].

Histology commonly reveals extensive proliferation of endothelial cells interstitially arranged around collagen bundles within the papillary and reticular dermis, forming small capillary vessels with barely visible lumina. It can be distinguished from RAE, in which the proliferation of endothelial cells occurs inside the capillary lumina of dermal vessels [7]. DDA can occasionally show proliferating cells forming small vascular channels. These cells may have a spindle-shaped appearance and a vacuolated cytoplasm [7]. Immunohistochemically, proliferating cells in the dermis express endothelial markers such as erythroblast transformation-specific related gene, CD31, and CD34, underscoring benign dermal endothelial cell proliferation [2,4,8]. Pericytes positive for alpha-smooth muscle actin (α-SMA) surround the new fully functioning vessels seen in DDA, emphasizing the benign aspect of this condition [2].

The differential diagnosis for DDA includes acroangiodermatitis (pseudo-Kaposi sarcoma), Kaposi sarcoma, low-grade angiosarcoma, and any occlusive vasculopathy/vasculitis causing retiform purpura [5,9]. DDA can be histologically distinguished from Kaposi sarcoma because the cellular atypia, frank spindling, inflammatory infiltration, and promontory signs seen in Kaposi sarcoma are not present in DDA [7]. Acroangiodermatitis is typically seen in the papillary dermis, with moderate endothelial proliferation in a lobular presentation, compared to DDA’s presence in the full dermis [7]. Angiosarcoma can be differentiated by atypia, mitoses, endothelial cell layering, nuclear hyperchromasia, and the absence of α-SMA-positive pericytes in the outer layer [2].

## Conclusions

We report this case because of the uncommon presentation of DDAB. DDAB typically manifests as reticulated erythematous to violaceous plaques with a tendency to centrally ulcerate and is exacerbated by hypoxic risk factors. Clinicians should suspect DDAB when older patients present with macromastia, atherosclerosis, and a history of smoking. The mainstay of treatment is the removal of inflammatory factors through lifestyle modification, such as smoking cessation. Reduction mammoplasty is a viable treatment option that can be considered in patients who fail to improve with conservative therapy. Our findings contribute to the understanding of the pathology and treatment of DDAB, particularly the role of removing inflammatory factors as a non-invasive approach to therapy.
